# High Diversity and Prevalence of *Borrelia burgdorferi* sensu lato in Wildlife Hosts, Domestic Animals, and Ticks in Yunnan Province, Southwestern China

**DOI:** 10.3389/fmicb.2022.876079

**Published:** 2022-05-04

**Authors:** Zhihai He, Baogui Jiang, Lin Huang, Zongti Shao, Yun Zhang, Yuqiong Li, Ennian Pu, Xingde Duan, Hang Jiang, Jian Wang, Mingguo Yao, Fan Wang, Shuangshuang Bie, Michael E. von Fricken, Yi Sun, Yi Dong, Jiafu Jiang, Chunhong Du

**Affiliations:** ^1^Yunnan Institute of Endemic Diseases Control and Prevention, Yunnan, China; ^2^Longgang Center for Disease Control and Prevention of Shenzhen, Guangdong, China; ^3^State Key Laboratory of Pathogen and Biosecurity, Beijing Institute of Microbiology and Epidemiology, Beijing, China; ^4^Department of Epidemiology and Biostatistics, School of Public Health, Anhui Medical University, Hefei, China; ^5^Department of Global and Community Health, George Mason University, Fairfax, VA, United States

**Keywords:** lyme disease, *Borrelia burgdorferi* sensu lato, small mammals, ticks, domestic mammals, China

## Abstract

*Borrelia burgdorferi* sensu lato (BBSL), the causative agent of Lyme disease, is commonly found in wild and domestic mammals and ticks worldwide. In China, human cases of *Borrelia burgdorferi* infections have been identified across a wide geographic range including Yunnan Province, but few studies have examined BBSL in reservoirs and vectors in southwestern China. Here we conducted a thorough and broad-range investigation of BBSL in small mammals, domestic mammals, and ticks collected from 159 sample sites across 42 counties in Yunnan Province. DNA was extracted from spleen tissue of small mammals, blood from domestic mammals, and homogenized ticks. Nested PCR targeting the 5S-23S rRNA intergenic spacer gene of BBSL was used for screening, with amplicons sequenced directly and analyzed using a BLAST algorithm. A total of 8,478 samples were collected, which were composed of 5,044 mammals belonging to 68 species, 1,927 livestock belonging to five species, and 1, 507 ticks belonging to 14 species. BBSL was detected in 147 mammals (2.9%) from 30 different species, 20 of which represent the first reported detection in that species. A total of 52 (2.7%) livestock samples were positive for BBSL, with dogs having the highest detection rate (6.3%, 43/687), and 103 ticks (6.8%) tested positive with high prevalence in *Ixodes granulatus* (44.2%, 23/52)*, Haemaphysalis nepalensi* (33.3%, 3/9) and *Haemaphysalis kolonini* (19.0%, 31/163). Sequence analysis revealed six genospecies of BBSL including *B. afzelii*, *B. burgdorferi sensu stricto*, *B. japonica*, *B. garinii*, *B. sinica,* and *B. valaisiana*. Significant differences in prevalence rates of BBSL were observed by species, landscape types, altitude, and season. Our findings indicate a wide distribution of multiple endemic BBSL genospecies based on a large-scale survey within Yunnan, which underline the need to expand surveillance efforts for human in southwestern China.

## Introduction

Lyme borreliosis (LB) is the most common reported tick-borne disease across Europe, North America, and Asia, which has become a worldwide public health problem and remains large challenges (Hao et al., 2011; Leeflang et al., 2016; Schwartz et al., 2017; Bobe et al., 2021; Vandekerckhove et al., 2021). The causative agents of LB fall within the species complex *Borrelia burgdorferi* sensu lato (BBSL) and are responsible for a wide spectrum of clinical symptoms including skin erythema, arthritis, and multi-system and multi-organ damage such as heart and nerve (Postic et al., 1994; Tamura et al., 2011). BBSL is transmitted by the bites of infected *Ixodes* tick, and main reservoir hosts include wild vertebrates such as rodents, deer and birds, and livestock animals, which take major contributors to the transmission of this zoonotic parasitosis across the globe (Hussain et al., 2021). More than 20 genospecies of BBSL have been described around the world, 10 of which have been reported to cause human infections (Hao et al., 2011; Hao, 2020).

In China, since LB was first found in Hailin County, Heilongjiang Province, in 1986, there have been documented reports of human cases of LB across almost all provinces in mainland China (Wu et al., 2013; Fang et al., 2015). The epidemic areas of Lyme disease in China are mainly concentrated in northeast, northwest, and parts of North China. However, the pathogen genospecies of most of LB cases were not characterized (Fang et al., 2015). Up to now, at least 100 strains belong to at least seven genotypes including *B. burgdorferi* sensu stricto, *B. garinii*, *B. afzelii*, *B. sinica, B. valaisiana*, *B. bavariensis,* and *B. bisettii* from patients, and ticks vector or host animals have been reported (Hao et al., 2016). Among of them, *B. garinii* and *B. afzelii* are the main pathogenic genotypes.

In Yunnan Province, positive serologies against BBSL in human and rodents have been reported over two decades ago (Wan et al., 1991; Pan and Yu, 1992). Other studies examining the prevalence of BBSL are relative lack (Hao et al., 2011; Hao, 2020). Yunnan Province is of particular interest given its wide topographic range and diverse land features including plateaus, low mountains, hills, high mountains, deep valleys, and open river valleys. A high level of small mammal and tick biodiversity exists in this region, often overlapping with largescale agricultural operations. To evaluate the distribution and genetic diversity of BBSL in reservoirs, incidental hosts, and vectors, we performed a broad-range, systematic field investigation of BBSL in Yunnan Province.

## Materials and Methods

### Sampling Protocol

**Figure 1:**
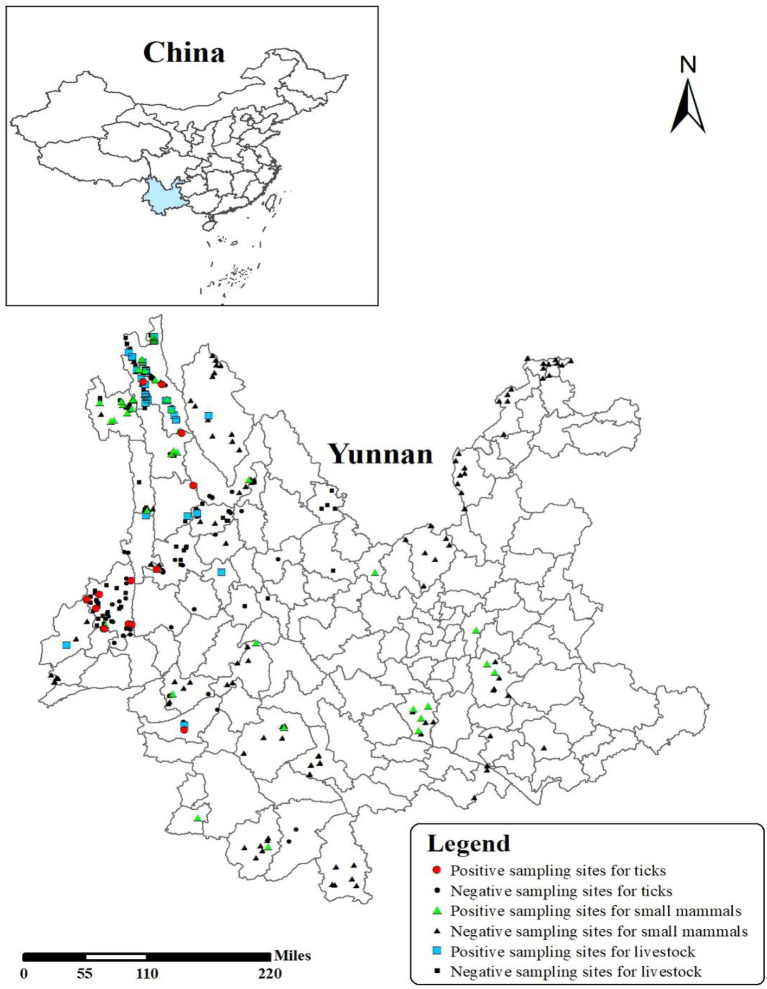
The distribution of sampling sites and prevalence of *Borrelia burgdorferi* sensu lato in Yunnan Province. Small mammals/live stocks/ticks were collected from 40 sample counties, including 21 positive sample counties (Tengchong, Deqin, Weixi, Gongshan, Shiping, Yongde, Fugong, Lanping, Gengma, Yiliang, Yunxian, Menglian, Yunlong, Mile, Shangri-La, Yulong, Jinggu, Menghai, Yingjiang, Jianchuan, and Yangbi).

During 2011–2021, small mammals were collected using animal snap traps set in agricultural, forested, and residential areas, with elevation ranging from 530 to 4,300 m, at a total of 159 sample sites located in 42 counties across Yunnan Province (Figure 1; Table 1). Mammal species were identified according to external morphology, fur color, measurements, and visible characters of dentition. After identification of species, spleen tissues were removed from the animals and stored in liquid nitrogen until tested, with engorged ticks opportunistically removed from the small mammals at same time. For unidentified species in the field, craniums were brought to the laboratory for further identification. Venous blood samples were concurrently collected from livestock in close proximity to a subset of sample sites using EDTA anticoagulant tubes, with engorged ticks opportunistically removed from livestock at same time. Questing ticks were actively collected from vegetation by flagging, followed by morphological identification under a stereomicroscope and then stored at −80°C.

**Table 1:** Prevalence of *Borrelia burgdorferi* sensu lato at different sampling sites.

Sampling counties	No. of positive/Tested (%)			
	Small mammals	Livestock	Ticks	Total
Deqin	52/677(7.7)	44/426(10.3)	6/70(8.6)	102/1173(8.7)
Weixi	8/239(3.3)	–	31/174(17.8)	39/413(9.4)
Yulong	1/224(0.4)	–	0/72(0.0)	1/296(0.3)
Gongshan	61/711(8.6)	0/120(0.0)	–	61/831(7.3)
Fugong	1/134(0.7)	1/139(0.7)	–	2/273(0.7)
Jinggu	1/76(1.3)	–	–	1/76(1.3)
Tengchong	1/39(2.6)	0/376(0)	47/405(11.6)	48/820(5.9)
Yongde	3/215(1.4)	–	0/6(0.0)	3/221(1.4)
Menghai	1/177(0.6)	–	–	1/177(0.6)
Yunxian	1/68(1.5)	–	–	1/68(1.5)
Shiping	6/12(4.7)	–	–	6/128(4.7)
Mile	2/110(1.8)	–	–	2/110(1.8)
Yiliang	6/94(6.4)	–	–	6/94(6.4)
Yunlong	2/177(1.1)	0/100(0.0)	1/127(0.79)	3/404(0.7)
Menglian	1/304(0.3)	–	–	1/304(0.3)
Jinping	0/27(0.0)	–	–	0/27(0.0)
Wenshan	0/17(0.0)	–	–	0/17(0.0)
Yingjiang	0/38(0.0)	1/109(0.9)	–	1/147(0.7)
Shangri-La	0/91(0.0)	2/209(1.0)	–	2/300(0.7)
Lushui	0/114(0.0)	–	0/40(0.0)	0/154(0.0)
Jianchuan	0/112(0.0)	1/115(0.9)	0/284(0.0)	1/511(0.2)
Ninger	0/88(0.0)	–	–	0/88(0.0)
Mengla	0/81(0.0)	0/26(0.0)	–	0/107(0.0)
Mengzi	0/22(0.0)	–	–	0/22(0.0)
Shuifu	0/129(0.0)	–	–	0/129(0.0)
Suijiang	0/93(0.0)	–	–	0/93(0.0)
Longchuan	0/170(0.0)	–	–	0/170(0.0)
Yongshan	0/97(0.0)	–	–	0/97(0.0)
Qiaojia	0/127(0.0)	–	–	0/127(0.0)
Lvquan	0/120(0.0)	–	–	0/120(0.0)
Yuanmou	0/228(0.0)	–	–	0/228(0.0)
Eryuan	0/117(0.0)	–	0/20(0.0)	0/137(0.0)
Lanping	–	1/30(3.3)	4/77(5.2)	5/107(4.7)
Gengma	–	1/34(2.9)	14/187(7.5)	15/211(7.1)
Yangbi	–	1/100(1.0)	–	1/100(1.00)
Huaping	–	0/103(0.0)	–	0/103(0.0)
Midu	–	0/9(0.0)	–	0/9(0.0)
Dayao	–	0/13(0.0)	–	0/13(0.0)
Weishan	–	0/18(0.0)	–	0/18(0.0)
Yongping	–	–	0/3(0.0)	0/3(0.0)
Heqin	–	–	0/14(0.0)	0/14(0.0)
Jinghong	–	–	0/28(0.0)	0/28(0.0)
Total	147/5044(2.9)	52/1927(2.7)	103/1507(6.8)	302/8478(3.6)

### DNA Extraction and PCR Analysis

DNA was extracted from spleen tissue of small mammals, livestock blood, and tick samples using the DNA blood and tissue kits (Tiangen Biotechnique, Beijing, China) according to the manufacturer’s instruction. A nested PCR for the 5S-23S rRNA intergenic spacer gene of BBSL using first-round primers (5′-CGACCTTCTTCGCC TTAAAGC-3′ and 5′-TAAGCTGACTAATACTAATTACCC-3′) and second round primers (5′-TCCTAGGCATTCACCATA-3′ and 5′-CTGCGAGTTCGCG GGAGA-3′) was performed as previously described (Postic et al., 1994). The PCR-positive amplicons were directly sequenced using an automated DNA sequencer (ABI PRISM 373; Perkin-Elmer, Norwalk, CT). Sequence analysis was carried out using a FASTA search of the GenBank database, with phylogenetic trees constructed using MEGA software, version 6.06 (Tamura et al., 2011). The sequences of BBSL obtained in this study were deposited in GenBank under accession numbers MK333406-MK33427, MZ146346, MZ146350, MZ146351, and OM350159-OM350185, respectively.

### Statistical Analysis

Univariate analysis was used to assess the association between gender, developmental stage of small mammals, environmental landscape, altitude, and positive detection rate of BBSL using a chi-square test. All variables with a value of *p* < 0.05 from univariate analysis were included in a multivariate forward stepwise logistic regression. All analyses were conducted using SPSS (version 17.0, SPSS Inc. Chicago, IL).

### Ethics Statement

The research protocol for trapping wild small animals and collecting samples was approved by the Animal Subjects Research Review Boards at the Yunnan Institute of Endemic Diseases Control and Prevention (2011-005), in accordance with the medical research regulations of China and the Regulation of the People’s Republic of China for the Implementation of the Protection of Terrestrial Wildlife. The efforts were made to avoid trapping in protected habitats.

## Results

A total of 5,044 small mammals, 1,927 livestock samples, and 1,507 ticks were collected, with 302 (3.6%) positive samples of BBSL detected in 21 of 42 sample counties (Figure 1; Table 1). Weixi County had the highest prevalence (9.4%), followed by Deqin (8.8%), and Gongshan (7.3%), with significant differences in positive rates of BBSL by counties observed (*χ*^2^ = 201.729, *p* < 0.05). Positive BBSL in small mammals were detected in 15 out of 32 sampled counties, with highest prevalence in Gongshan County (8.6%). The positive sampling points of BBSL in livestock were detected in 8 out of 16 sampling counties with the highest prevalence in Deqin (10.3%). *Borrelia burgdorferi* s.l. in ticks were detected from 6 out of 14 sampling counties with the highest prevalence in Weixi County (17.8%; Table 1).

### The Prevalence of BBSL in Small Mammals

A total of 68 species of small mammals were tested in this study, with details provided in Table 2. *Rattus flavipectus* was the most common species captured (16.5%, 833/5044), followed by *Apodemus draco* (11.5%, 582/5044). A total of 147 (2.9%) small mammals tested positive, with *Ochotona gloveri* (33.3%, 1/3), *Soriculus leucops* (14.6%, 13/89), *R. tuekkestanicus* (14.3%, 1/7), and *Sorex cylindricauda* (13.0%, 7/54) actively infected with BBSL. Five different genotypes of BBSL were found, with *Borrelia afzelii* (56.5%, 83/147) being the highest proportion of positive detections in small mammals, followed by *B. garinii* (20.4%, 30/147)*, B. burgdorferi sensu stricto* (18.3%, 27/147)*, B. valaisiana* (3.4%, 5/147), and *B. japonica* (1.4%, 2/147)*. Borrelia afzelii* was detected in 24 different species of small mammals with 1.6% (83/5044) detections overall, with *A. chevrrieri* (19.3%,16/83), *A. draco* (12.0%, 10/83), and *Eothenomvs Eleusis* (9.6%, 8/83) as the most common reservoirs. The detection rate of *B. garrinii* in small mammals was 0.6% (30/5044) and found mainly in *A. lartomm* (23.3%, 7/30) and *Niviventer confucianus* (23.3%, 7/30). Details on the limited detections of *B. valaisiana* (0.1%, 5/5044) and *B. japonica* (0.04%, 2/5044) by host can be found in Table 2. Of note, *Apodemus draco*, *A. chevrieri*, *A. latronum,* and *Niviventer confucianus* species tested positive for three or more genotypes of BBSL.

**Table 2:** Prevalence of *Borrelia burgdorferi* sensu lato in different species.

Species	Positive/Test (%)	*B.* *afzelii*	*B.* *burgdorferi*	*B.* *garinii*	*B.* *valaisiana*	*B.* *japonica*	*B.* *sinica*
**Small mammals**							
*Rattus flavipectus*	4/833(0.5)	3	0	0	1	0	0
*Rattus tuekkestanicus*	1/7(14.3)	1	0	0	0	0	0
*Rattus norvegicus*	2/97(2.1)	1	1	0	0	0	0
*Rattus brunneusculus*	3/275(1.1)	1	0	0	2	0	0
*Apodemus latronum*	9/174(5.2)	1	1	7	0	0	0
*Apodemus chevrieri*	20/475(4.2)	16	1	3	0	0	0
*Apodemus draco*	19/582(3.3)	10	5	3	0	1	0
*Mus caroli*	3/100(3.0)	3	0	0	0	0	0
*Mus pahari*	6/133(4.5)	6	0	0	0	0	0
*Niviventer andersoni*	3/57(5.3)	0	0	2	1	0	0
*Niviventer eha*	2/32(6.3)	0	2	0	0	0	0
*Niviventer confucianus*	14/220(6.4)	6	1	7	0	0	0
*Niviventer excelsior*	1/29(3.4)	0	0	0	0	1	0
*Eothenomys eleusis*	12/160(7.5)	8	4	0	0	0	0
*Eothenomys cachinus*	4/38(10.5)	3	1	0	0	0	0
*Eothenomys custos*	2/95(2.1)	1	1	0	0	0	0
*Pitymys leucurus*	1/48(2.1)	0	0	1	0	0	0
*Volemys clarkei*	2/35(5.7)	1	0	1	0	0	0
*Dremomys pernyi*	3/30(10.0)	0	0	3	0	0	0
*Crocidura attenuata*	2/77(2.6)	1	0	0	1	0	0
*Crocidura dracula*	1/79(1.3)	1	0	0	0	0	0
*Soriculus caudatus*	1/46(2.2)	1	0	0	0	0	0
*Soriculus leucops*	13/89(14.6)	6	7	0	0	0	0
*Sorex alpinus*	1/30(3.3)	1	0	0	0	0	0
*Sorex cylindricauda*	7/54(13.0)	6	1	0	0	0	0
*Anourosorex squamipes*	2/146(1.4)	0	2	0	0	0	0
*Suneus murinus*	1/163(0.6)	1	0	0	0	0	0
*Nasillus gracilis*	1/39(2.6)	1	0	0	0	0	0
*Ochoto thibeta -*	6/92(6.5)	3	0	3	0	0	0
*Ochoto gloveri*	1/3(33.3)	1	0	0	0	0	0
Others	0/806(0.0)	0	0	0	0	0	0
Total	147/5044(2.9)	83	27	30	5	2	0
**Livestock**							
Bovine	5/554(0.9)	5	0	0	0	0	0
Sheep	3/636(0.5)	1	2	0	0	0	0
Dog	43/687(6.3)	40	2	1	0	0	0
Horse	1/33(3.0)	1	0	0	0	0	0
Donkey	0/17(0.0)	0	0	0	0	0	0
Total	52/1927(2.7)	47	4	1	0	0	0
**Ticks**							
*I. granulatus*	23/52(44.2)	0	0	0	22	1	0
*Ha. kolonini*	31/163(19.0)	2	0	29	0	0	0
*I. ovata*	34/317(10.7)	7	1	10	0	0	16
*R. microplus*	12/624(1.9)	4	0	8	0	0	0
*Ha. nepalensis*	3/9(33.3)	1	0	2	0	0	0
*Ha. pitini*	0/14(0)	0	0	0	0	0	0
*I. acuminata*	0/17(0)	0	0	0	0	0	0
*D. auratus*	0/7	0	0	0	0	0	0
*R. haemaphysaloides*	0/64	0	0	0	0	0	0
*H. longicornis*	0/5	0	0	0	0	0	0
*H. montgomeryi*	0/201	0	0	0	0	0	0
*H. yeni*	0/14	0	0	0	0	0	0
*A. testudinarium*	0/10	0	0	0	0	0	0
*H. menglaensis*	0/10	0	0	0	0	0	0
Total	103/1507(6.8)	14	1	49	22	1	16
In all in total altogether	302/8478(3.6)	144	32	80	27	3	16

### The Prevalence of BBSL in Livestock

A total of 1927 livestock was composed of 554 bovines, 636 sheep, 687 dogs, 33 horses, and 17 donkeys. Among them, 52 were positive for BBSL, with dogs having the highest prevalence (6.3%, 43/687), followed by horses (3.0%, 1/33), bovines (0.9%, 5/554), and sheep (0.5%, 3/636). Three BBSL genotypes were detected, including 47 *B. afzelii*, four *B. burgdorferi sensu stricto*, and one *B. garinii*. The dominant *B. afzelii* genotype was primarily found in dogs (85.1%, 40/47), followed by bovines (10.6%, 5/47), sheep (2.1%, 1/47), and horses (2.1%, 1/47; Table 2).

### The Prevalence of BBSL in Ticks

A total of 103 out of 1, 507 ticks belonging to 14 species tested positive for BBSL. Species-specific detections are as follows: *Ixodes granulatus* 44.2% (23/52)*, Haemaphysalis nepalensi* 33.3% (3/9), *Haemaphysalis kolonini* 19.0% (31/163), *I. ovata* 10.7% (34/317), and *R. microplus* 1.9% (12/624), respectively. Six known BBSL genotypes were found including *B. garinii* (47.6%, 49/103), *B. valaisiana* (21.4%, 22/103), *B. sinica* (15.5%, 16/103), *B. afzelii* (13.6%, 14/103), *B. burgdorferi sensu stricto* (1.0%, 1/103), and *B. japonica* (1.0%, 1/103)*. Borrelia afzelii* was primarily detected in *I. ovata* (50.0%, 7/14) and *R. microplus* (28.6%, 4/14). Both *B. valaisiana* and *B. japonica* were only detected in *I. granulatus* with low prevalence, which aligns with small mammal test results. *Borrelia sinica*, which was not found in small mammals and livestock samples, was detected in 1.1% (16/1507) of *I. ovata* ticks.

### The Distribution of BBSL Genotypes by Sample Sites

As shown in Table 3, the proportion of *B. afzelii* genotype of BBSL in positive samples was highest, 47.7% (144/302) in Deqin (43.8%, 63/144), Gongshan (27.1%, 39/144), and Tengchong (6.9%, 10/144). *Borrelia burgdorferi sensu stricto* was primarily detected in Gongshan (68.8%, 22/32) and Deqin (9.4%, 3/32). *Borrelia garinii* was also detected in Deqin (43.8%, 35/80), Weixi (36.3%, 29/80), and Tengchong (16.3%, 13/80). One unidentified *Borellia* sp. was found in Tengchong. Additional details on geographic distribution of BBSL by sites for *B. valaisiana, B. japonica*, and *B. sinica* can be found in Table 3. Deqin County had a distribution of four *Borellia* spp. excluding *B. japonica* which was only found in Yunlong County (Table 3).

**Table 3:** Results of Genotypes of BBSL in 21 positive sampling counties.

**Sampling sites**	**Positive/Tested** **(%)**	***B.*** ***afzelii***	***B.*** ***burgdorferi***	***B.*** ***garinii***	***B.*** ***valaisiana***	***B.*** ***japonica***	***B.*** ***sinica***
Deqin	102/1158 (8.8)	63	3	35	1	0	0
Weixi	39/413 (9.4)	8	2	29	0	0	0
Yulong	1/296 (0.3)	1	0	0	0	0	0
Gongshan	61/831 (7.3)	39	22	0	0	0	0
Fugong	2/273 (0.7)	1	1	0	0	0	0
Jinggu	1/76 (1.3)	1	0	0	0	0	0
Tengchong	48/820 (5.9)	10	0	13	23	0	2
Yongde	3/221 (1.4)	2	0	0	1	0	0
Menghai	1/177 (0.6)	1	0	0	0	0	0
Yunxian	1/68 (1.5)	0	0	0	1	0	0
Shiping	6/128 (4.7)	5	1	0	0	0	0
Mile	2/110 (1.8)	2	0	0	0	0	0
Yiliang	6/94 (6.4)	6	0	0	0	0	0
Yunlong	3/404 (0.7)	0	0	0	0	3	0
Menglian	1/304(0.3)	0	0	0	1	0	0
Yingjiang	1/147(0.7)	1	0	0	0	0	0
Shangri-La	2/315 (0.6)	2	0	0	0	0	0
Jianchuan	1/511 (0.2)	1	0	0	0	0	0
Lanping	5/107 (4.7)	0	2	3	0	0	0
Gengma	15/221 (6.8)	0	1	0	0	0	14
Yangbi	1/100 (1.0)	1	0	0	0	0	0
Total	302/6774 (4.5)	144	32	80	27	3	16

### The Temporal Distribution of BBSL in Yunnan

**Figure 2:**
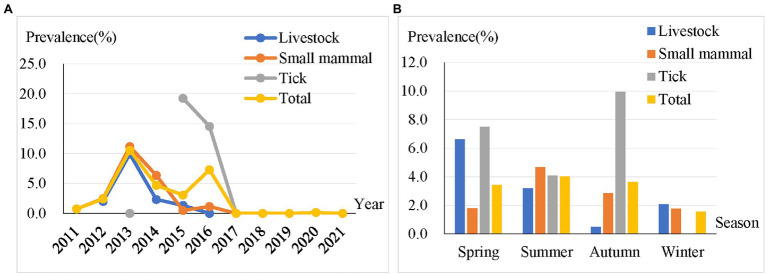
Prevalence of BBSL in different years **(A)** and four seasons **(B)** for livestock, small mammals, ticks, and total samples.

We examined the temporal distribution of a total of 302 positive samples. The results showed that BBSL in livestock, small mammals, and ticks dominated in the years 2013 (9.9%), 2013 (11.2%), and 2015 (19.3%), respectively (Figure 2A). The highest prevalence of BBSL was also observed in the spring for livestock (6.6%), summer for small mammals (4.7%), and autumn for ticks (10.0%). It appears that there was no significant different in the four seasons among the prevalence of BBSL carried by livestock, small mammals, and ticks throughout the entire study (Figure 2B).

### Phylogenetic Analyses

**Figure 3:**
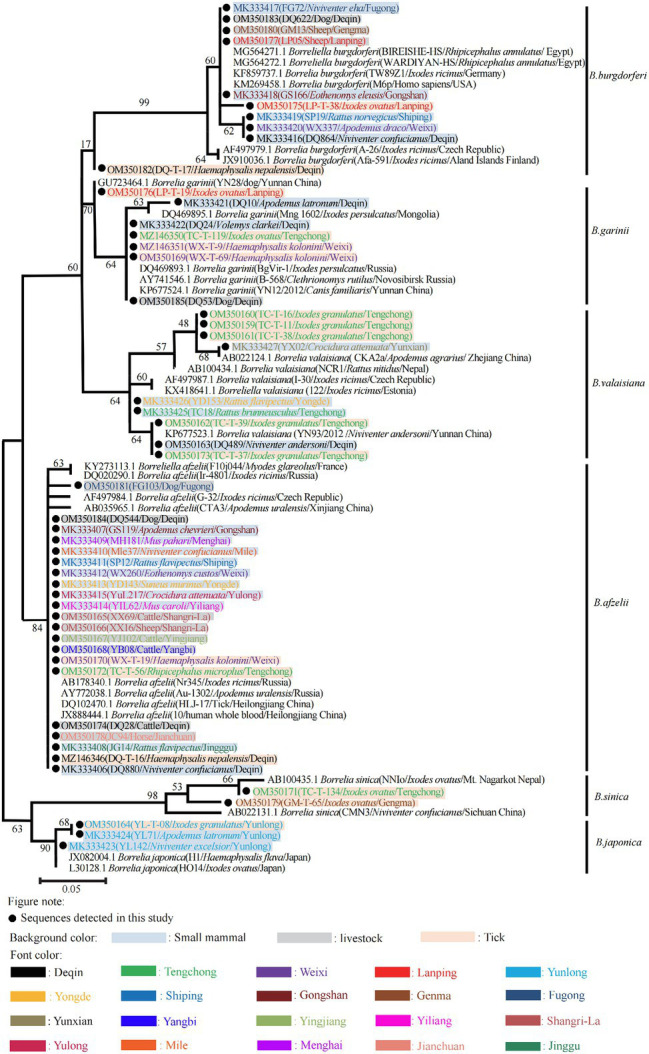
Phylogenetic tree of 252 bp of 5S-23S rRNA intergenic spacer gene. Maximum likelihood phylogenetic tree based on a comparison of *Borrelia burgdorferi* sensu lato 5S-23S rRNA intergenic spacer gene sequences obtained from Yunnan small mammals/live stocks/ticks with *Borrelia burgdorferi* sensu lato reference strains. The number on each branch shows the percent occurrence in 5000 bootstrap replicates. Black circles stood for sequences identified in this study.

Phylogenetic analyses based on different representative sequences in this study determined that all detected *Borrelia* spp. fell within six separate clades belonging to six different known types of BBSL including *B. afzelii*, *B. burgdorferi sensu stricto*, *B. garinii*, *B. japonica*, *B. sinica,* and *B. valaisiana* (Figure 3). The nucleotide sequences of *B. afzelii* isolates were closely related to the BBSL isolated from a patient in China (JX888444.1). All *B. burgdorferi sensu stricto* sequences were identical to the strain BRE-13 isolated from a patient’s cerebrospinal fluid in France (KY594010.1). *Borrelia garinii* sequences detected in this study shared 99% identity with the strain YN12/2012 detected from *Canis familiaris* in Yunnan Province. *Borrelia japonica* sequences shared 99% identity with strain Cow611C from a tick in Japan (L30125.1). The *B. valaisiana* sequences were 98% similar to the strain KM2 from *Ixodes granulatus* ticks in Taiwan, China (HM100110.1), and 98% similar to the strain CKA2a from *A. agrarius* in Zhejiang Province, China (AB022124.1).

### Risk Factors Related to *Borrelia burgdorferi* sensu lato Infection

Significant differences in detection rates of BBSL in small mammals, livestock, and ticks were observed (*χ*^2^ = 57.329, *p* < 0.001). Further stratified analysis by species suggests that the prevalence of BBSL in small mammals and tick species, livestock, and tick species was also significant (*χ*^2^ = 48.586, *p* < 0.017; *χ*^2^ = 33.569, *p* < 0.017), respectively. A significant difference of BBSL prevalence in small mammals was observed by altitude class (<1,500 m, 1,500–2,500 m, >2,500 m), with 0.5, 1.8, and 5.4% detected prevalence, respectively (*χ*^2^ = 81.728, *p* < 0.001), as well as by landscape type with forested landscapes 4.4%, agricultural landscapes 2.1%, and residential landscapes 0.3%, respectively (*χ*^2^ = 29.992, *p* < 0.001; Table 4). Multivariate logistic regression analysis also suggests that samples found at altitudes 1,500 m ~ 2,500 m and >2,500 m were more likely to be infected with BBSL (OR 3.410, 95% CI 1.527 ~ 7.616, *p* = 0.003; OR 10.693, 95%CI 5.206 ~ 21.962, *p* < 0,001). In addition, the positive detection rates of BBSL in free ticks and parasitic ticks were different, with 13.0 and 4.7%, respectively (*χ*^2^ = 30.738, *p* < 0.001). And the ticks removed from different hosts (small mammals and livestock) were also different, with 36.0 and 3.3%, respectively (*χ*^2^ = 106.587, *p* < 0.001). A significant difference in BBSL detections in ticks was also observed by landscape types (forest, agriculture and residential), with 4.7, 25.0, and 10.1%, detected prevalence, respectively (*χ*^2^ = 58.323, *p* < 0.001; Table 4).

**Table 4:** Risk factors related to *Borrelia burgdorferi* sensu lato based on univariate analyses.

Stratified analysis variable		Sample size	BBSL infection		
		Constituent ratio (%)	Positive rate (%)	*χ* ^2^	*P*
**Samples type**					
	small mammals	5044/8478(59.5)	147/5044(2.9)	57.329	<0.001
	livestock	1927/8478(22.7)	52/1927(2.7)		
	ticks	1507/8478(17.8)	103/1507(6.8)		
**Small mammals**	Altitude (m)				
	~1,500	1521/5025(30.2)	8/1521(0.5)		
	1,500 ~ 2,500	1355/5025(27.0)	24/1355(1.8)	81.728	<0.001
	2,500~	2149/5025(42.8)	115/2149(5.4)		
	Gender				
	male	2415//5025(48.1)	82/2415(3.4)	3.618	0.057
	female	2610//5025(51.9)	65/2610(2.5)		
	Age				
	adult	4398//5025(87.5)	134/4398(3.0)	1.831	0.176
	pubertal	627/5025(12.5)	13/627(2.1)		
	Landscape				
	residential	306//5025(6.1)	1/306(0.3)		
	agricultural	2684/5025(53.4)	56/2684(2.1)	29.992	<0.001
	forest	2035//5025(40.5)	90/2035(4.4)		
**Ticks**	Altitude (m)				
	~1,500	85/1507(5.6)	23/85(27.1)		
	1,500 ~ 2,500	737/1507(48.9)	41/737(5.6)	57.871	<0.001
	2,500~	685/1507(45.5)	39/685(5.7)		
	Parasitic hosts				
	small mammal	50/1122(4.5)	18/50(36.0)	106.587	<0.001
	livestock	1072/1122(95.5)	35/1072(3.3)		
	Parasitism				
	free	385/1507(25.5)	50/385(13.0)	30.738	<0.001
	parasitic	1122/1507(74.5)	53/1122(4.7)		
	Age				
	adult tick	1419/1507(94.2)	100/1419(7.0)	1.722	0.274
	pubertal tick	88/1507(5.8)	3/88(3.4)		
	Gender				
	male	498/1419(35.1)	24/498(4.8)	5.814	0.016
	female	921/1419(64.9)	76/921(8.3)		
	Landscape				
	forest	1151/1507(76.4)	54/1151(4.7)		
	agricultural	88/1507(5.8)	22/88(25.0)	58.323	<0.001
	residential	268/1507(17.8)	27/268(10.1)		

## Discussion

Human cases of LB have been confirmed in almost every province found on mainland China, including Yunnan Province. However, most reported patients only have serological evidence indicating previous LB infection, without confirmed specific genotypes information. BBSL have been reported in small mammals trapped in the provinces Qinghai, Hunan, Shanxi, Liaoning, Sichuan, Fujian, Zhejiang, Gansu, Guangdong, Jilin, and Yunnan (Wan et al., 1991; Pan and Yu, 1992; Chen et al., 2000; Shi et al., 2004; Huang et al., 2006; Hou et al., 2010; Meng et al., 2010; Wu et al., 2013), indicating that small mammals are likely the main reservoir hosts in China. This study presents a much larger context of BBSL in Yunnan, including sampling both small mammals, livestock, and ticks across a wide and diverse geographic area. The findings from this survey provide valuable insights about the prevalence, spatial distribution, and genetic diversity of BBSL in Yunnan Province, southwestern China.

We documented BBSL infection in 30 species of small mammals, among which 20 species had not been previously described as potential reservoirs. It is possible these 20 species are incidental hosts that are infected occasionally, requiring additional investigations to determine what, if any, role they play as reservoir hosts. The *R. tanezumi* (16.5%) was the predominant species trapped in residential areas in Yunnan. *Apodemus* spp. (24.7%) were the predominant hosts captured in Yunnan, which is consistent with findings from Europe where *Apodemus* are considered a major reservoir of *Borrelia* (Sala and De, 2016). *Borrelia burgdorferi* s.l. was also detected in *A. draco* and *A. chevrieri* in Yunnan, with *A. draco* capable of carrying four pathogenic *Borrelia* spp. *Soriculus leucops* had a much higher prevalence (>14.0%) detected in this study, when compared to other provinces in China (Wang et al., 2003; Chu et al., 2006; Huang et al., 2006; Zhang et al., 2015, 2016; Wulantuya and Yin, 2018). *Rattus tuekkestanicus*, a prominent household species in Yunnan, had a relatively high prevalence (14.3%), although only 7 small mammals were captured. We also found that *Sorex cylindricauda* tested positive for BBSL DNA (13.0%), requiring further investigation to fully understand their role in maintaining or amplifying infections in nature.

In this study, 52 positive livestock blood samples were detected in 8 counties which were mainly distributed in western Yunnan Province. Cattle, sheep, dogs, and horses were detected positive, with this study reporting the first ever detections of BBSL in Yunnan in cattle, sheep, and horses. The infection rate in dogs was the highest, with three known BBSL genotypes detected including *B. afzelii*, *B. burgdorferi* s.s., and *B. garinii*, which are all pathogenic in humans. Tick samples from 6 out of 9 counties were positive, mainly distributed in the western of Yunnan Province. Six genotypes of *Borrelia burgdorferi* (*B. afzelii*, *B. garinii*, *B. japonica*, *B. valaisiana*, *B. burgdorferi sensu stricto*, and *B. sinica*) were detected from five tick species (*Ha. nepalensis*, *I. ovatus*, *I. granulatus*, *R. microplus,* and *Ha. kolonini*), with *B. garinii* as the dominant species (47.6%,49/103) and *R. microplus* was the dominant ticks (41.4%, 624/1507). This study is the first report of *B. japonica* detected in *I. granulosus* in China*. Borrelia sinica* was also detected in *I. ovata* for the first time in Yunnan Province.

Both *Borrelia afzelii* and *B. garinii* were detected in livestock, small mammals, and ticks in Deqin County, *B. japonica, B. valaisiana,* and *B. afzelii* were detected in small mammals and ticks in Yunlong, Tengchong, and Weixi County. There are more than three genotypes of BBSL in these three counties located western Yunnan, which warrants further investigation.

Our findings also indicated that detection rates in small mammals were ranked highest to lowest by landscape type as follows: forest landscape > agricultural landscape > residential landscape, which is likely related to vector density and preferred reservoir habitat. Sampling locations in this survey contained a broad range of altitudes from 500 to 4,500 m. Among the three altitude classes, the highest prevalence of BBSL was found above 2,500 m. In the Czech Republic, the distribution of *Ixodes ricinus,* a known vector of LB, extended toward higher altitudes, which was attributed to warming climates (Daniel et al., 2003). The roles temperature and humidity play in tick reproduction and reservoir preferences require further investigation within these altitude ranges. Additionally, there are no reported human cases at these heights, which might reflect lower populations living in these areas.

No obvious season-associated prevalence of BBSL in the host and vector beside winter was observed, which is different from those in Northern China, where there are four distinct season and temporal dynamic change of BBSL. We also observed the temporal dynamic change *via* years for BBSL, especially no positive samples after 2017. It is probably because of the deviation in sampling arrangement that most of samples were collected in the sites located northeastern Yunnan and Jinshajiang river low-altitude areas. It also coincides with our general finds that the prevalence of BBSL in Yunnan was mainly in high elevations in the west and northwest areas.

Six genospecies of BBSL were detected in this study, with four of them, excluding *B. japonica* and *B. sinica,* previously associated with clinical LB (Diza et al., 2004; Rudenko et al., 2011). The BBSL genotypes with the highest proportion of BBSL positive samples in small mammals, livestock, and ticks were *B. afzelii* and *B. garinii*, respectively. There exists a wide distribution and genetic diversity of BBSL in Yunnan, compared to only 1–2 genospecies of BBSL detected in other provinces of China; however, this may also be an artifact of the extent and volume of samples tested in this study. *Borrelia afzelii* was the predominant genotype (56.5%, 83/147) and was detected in 24 species. According to the sequences detected in this study, most *B. afzelii* isolates shared 99% identity with clinical samples from northeastern China (Ni et al., 2014), and *Borrelia garinii,* the most common genospecies elsewhere in China (Fang et al., 2015), was only detected in Deqin County in this study. While *B. burgdorferi sensu stricto* has previously been detected in *Cervus nippon* (Sika deer) from Jilin Province and in *Caprolagus sinensis* from Hunan Province, this study adds a number of detections in small mammals to the literature. *Borrelia valaisiana* sequences clustered into two clades, with one sharing sequence identity from Guizhou and Zhejiang Province and the other closer to sequences from Europe. While birds are major reservoirs for *B. valaisiana* in Europe, the transmission cycle maintaining *B. valaisiana* in Yunan may be different given findings from this study. Finally, *B. japonica* was detected in Yunlong County, which represents the first documentation of *B. japonica* in *A. draco* and *Niviventer excelsior* in China. At this time, there have been no confirmed patients with PCR confirmed LB in Yunnan Province, requiring further investigation.

## Conclusion

Yunnan Province is an important natural focus of BBSL in China. These findings reflect a high level of *Borrelia* genetic diversity found in a wide range of small mammals, many of which are likely reservoirs for BBSL in Yunnan. Given the absence of reported human cases within this region, efforts to expand clinical surveillance are needed.

## Data Availability Statement

The datasets presented in this study can be found in online repositories. The names of the repository/repositories and accession number(s) can be found at: MK333406-MK33427, MZ146346, MZ146350, MZ146351, and OM350159-OM350185.

## Author Contributions

CD, JJ, and YD conceived and designed the experiments. ZH, BJ, YZ, MY, FW, and SB performed the experiments. JJ, ZH, LH, BJ, and YS analyzed the data. Sample collections were implemented by ZS, XD, HJ, JW, YL, FW, SB, and EP. JJ, ZH, LH, and MF drafted and reviewed the manuscript. All authors contributed to the article and approved the submitted version.

## Funding

This study was funded by the National Key R&D Program (2019YFC1200501), the Natural Science Foundation of China (U2002219, 81760607, and 81360413), and the State Key Laboratory of Pathogen and Biosecurity (SKLPBS1833). The funders had no role in the study design, data gathering, analysis, interpretation, or writing of the paper. The corresponding authors had full access to all the data in the study and had final responsibility for the decision to submit for publication.

## Conflict of Interest

The authors declare that the research was conducted in the absence of any commercial or financial relationships that could be construed as a potential conflict of interest.

## Publisher’s Note

All claims expressed in this article are solely those of the authors and do not necessarily represent those of their affiliated organizations, or those of the publisher, the editors and the reviewers. Any product that may be evaluated in this article, or claim that may be made by its manufacturer, is not guaranteed or endorsed by the publisher.

## Abbreviations

BBSL: *Borrelia burgdorferi* sensu lato
LB: Lyme Borreliosis
PCR: Polymerase Chain Reaction
OR: Odds Ratio
95% CI: 95% Confidence Interval
